# Clonality and Micro-Diversity of a Nationwide Spreading Genotype of *Mycobacterium tuberculosis* in Japan

**DOI:** 10.1371/journal.pone.0118495

**Published:** 2015-03-03

**Authors:** Takayuki Wada, Tomotada Iwamoto, Aki Tamaru, Junji Seto, Tadayuki Ahiko, Kaori Yamamoto, Atushi Hase, Shinji Maeda, Taro Yamamoto

**Affiliations:** 1 Department of International Health, Institute of Tropical Medicine, Nagasaki University, Nagasaki, Japan; 2 Department of Microbiology, Kobe Institute of Health, Kobe, Japan; 3 Department of Microbiology, Osaka Prefectural Institute of Public Health, Osaka, Japan; 4 Department of Microbiology, Yamagata Prefectural Institute of Public Health, Yamagata, Japan; 5 Department of Microbiology, Osaka City Institute of Public Health and Environmental Sciences, Osaka, Japan; 6 Department of Mycobacterium Reference and Research, the Research Institute of Tuberculosis, Tokyo, Japan; University of Padova, Medical School, ITALY

## Abstract

*Mycobacterium tuberculosis* transmission routes can be estimated from genotypic analysis of clinical isolates from patients. In Japan, still a middle-incidence country of TB, a unique genotype strain designated as ‘M-strain’ has been isolated nationwide recently. To ascertain the history of the wide spread of the strain, 10 clinical isolates from different areas were subjected to genome-wide analysis based on deep sequencers. Results show that all isolates possessed common mutations to those of referential strains. The greatest number of accumulated single nucleotide variants (SNVs) from the oldest coalescence was 13 nucleotides, indicating high clonality of these isolates. When an SNV common to the isolates was used as a surrogate marker of the clone, authentic clonal isolates with variation in a reliable subset of variable number of tandem repeat (VNTR) genotyping method can be selected successfully from clinical isolates populations of *M*. *tuberculosis*. When the authentic clones can also be assigned to sub-clonal groups by SNVs derived from the genomic comparison, they are classifiable into three sub-clonal groups with a bias of geographical origins. Feedback from genomic analysis of clinical isolates of *M*. *tuberculosis* to genotypic markers will be an efficient strategy for the big data in various settings for public health actions against TB.

## Introduction

*Mycobacterium tuberculosis*, the etiological agent of tuberculosis (TB), still threatens public health of human beings. The huge burden it imposes on developing countries has derived not only from severe health problems in such areas but also from penetration to low-incidence areas through immigration. Acquisition of high levels of drug resistance of the bacilli is crucially important, prompting repeated warnings from WHO [1]. The bacilli spread drastically by diffusion of aerosol from the respiration of patients [2]. Detection and cutting of transmission routes is extremely important as an efficient action to control the expansion of TB.

Genotyping of *M*. *tuberculosis* clinical isolates is a powerful tool to identify the origins of infection in various cases [3,4]. Identical genotypes of isolates provide clues to the estimation of transmission among patients [3–5]. To increase the reliability of such estimation, the difference or identity of genotypes must be accurate. The discriminatory power of genotyping is necessary for practical use to ascertain the underlying pattern of transmission from surveillance studies. Variable number of tandem repeats (VNTR) genotyping is a popular method of genotyping [6] that has been widely used not only as a standard for global comparison but also as a practical tool for the strict discrimination of clinical isolates in local settings [5,7–10]. Optimized subsets for local usage are often adjusted to the phylogenetic property of *M*. *tuberculosis* in each area [11–13].

Generally, according to data accumulation, isolate clusters having identical genotypes tend to increase in the isolate population [14]. Concordance of genotypes not only provides supportive clues revealing transmission routes. It also elucidates inscrutable relations among patients without epidemiological links. In such cases, much more refined discrimination is needed to verify the true history of transmission. Recently, genome-wide analysis based on deep sequencers has been widely noticed to detect minute differences among clinical isolates of *M*. *tuberculosis* [15–22]. The accumulation of mutations in a series of clinical isolates can indicate the direction of transmission routes, even in outbreak cases [17,18]. Although the techniques are still costly, they have gradually become popular and constitute a standard tool for such public health purposes.

Japan, as an economically developed country located at the far eastern end of the Eurasian continent, has remained as a middle-prevalence area of TB (16.7/100,000 in 2012) [23]. The current situation might result from recurrence of elderly patients who lived during times of much severer prevalence (e.g., nearly 400/100,000 in 1961) [24]. Such patients also underscore the extreme *graying of society* prevailing in Japan. In terms of public health, it is important to differentiate sporadic recurrent cases of the current spread of bacilli to prevent the spread of TB in present situations. For the detection of transmission routes, surveillance based on a localized subset of VNTR genotyping has been introduced in Japan [12,25–27]. In addition, 24 loci of VNTR including highly polymorphic loci [8] have been incorporated to ascertain minute differences among isolates in surveillance trials.

Actually, the ‘M-strain’ was first reported as a causative strain of a large outbreak in the Tokyo metropolitan area in 2004 [28]. This strain, belonging to the modern Beijing sublineage, initially showed streptomycin resistance and an identical genotype based on various typing methods, such as restriction fragment length polymorphism (RFLP) [28] and VNTR genotypings [29]. In contrast to transitory outbreaks, the genotype has been identified from various areas with no continuous epidemiological links among patients [29,30]. The underlying reasons for its nationwide emergence remain unknown.

In this study, to estimate the background of the emergence, genotypically identical M-strains from patients with various circumstances such as geographical origins, household transmission, and acquisition of drug resistance in a patient were scrutinized using genomic comparison. Variations in the genomes were also verified as genotypic tools to refine the direction and scale of transmission of the strain in surveillance studies.

## Materials and Methods

### M-strains used for comparative genomics

**Fig 1:**
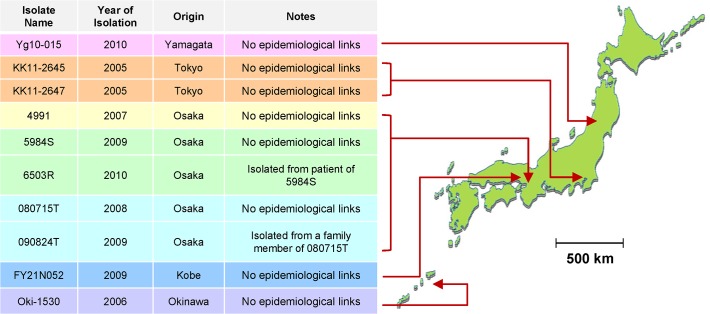
Profiles of 10 M-strain isolates subjected to genomic comparison. Locations of the origins are pointed on a geographical map of Japan.

For genomic comparison, 10 M-strains isolated from five cities in Japan (Yamagata, Tokyo, Osaka, Kobe, and Okinawa) were selected for examination in this study. They were isolated from culture stocks of *M*. *tuberculosis* clinical isolates by each local institute of public health for respective purposes such as surveillance, drug susceptibility tests, and contact tracing. The definition of M-strains is the following: streptomycin resistance, an identical genotype of 24 loci of VNTR [8] (S1 Table), and its sublineage (modern Beijing sublineage) [31,32]. The 24 VNTR loci were composed of Supply’s 15 standard [6], 12 of JATA, which was designed for discrimination of Beijing lineage strains [12], and 5 of hyper-variable loci (QUB-3232, VNTR 3820, VNTR 4120, QUB-11a, and QUB-18). The areas and years of isolation are presented in Fig. 1.

Epidemiologically, two isolates, 090824T and 080715T, from Osaka were isolated from household contacted patients, which were strongly regarded as linked with a single transmission. Another set of two isolates (5984S and 6503R) was isolated from an individual patient within 6 months. In this patient, the latter isolate was altered to iatrogenic MDRTB (rifampicin, isoniazid, and streptomycin resistant), although the former isolate was resistant only to streptomycin. Epidemiological links between the isolates and 4991 could not be found by inquiring survey from the patient, although it was isolated in Osaka too. It have been verified that two isolates from Tokyo (KK11-2645 and KK11-2647) also had no epidemiological links, although they were isolated from close areas in the same year. Patients of remaining three isolates (Yg10-015, FY21N052, and Oki-1530) were isolated from distant prefectures in different year, which had no contact with other patients of these isolates.

### Comparative genomics

Genomic DNAs of the 10 M-strains were purified as explained in an earlier report [33]. They were sequenced on the Illumina platform to obtain short pair-end reads (75 bp per read) using a Genome Analyzer IIx [DDBJ:PRJDB2911] (S2 Table). As an outgroup, short read sequences of T85 [DDBJ:SRX450070], a strain belonging to modern Beijing subfamily in a worldwide project [34,35], were retrieved. Using a Burrows-Wheeler Aligner (BWA) ver. 0.5.8c [36], 11 short read data were subjected to mapping analysis to the complete genome sequences of H37Rv [GenBank: AL123456.2] and CCDC5079 [GenBank:CP001641.1]. Substitution differences to the references were called by Genome traveler (In silico biology, Inc. Tokyo, Japan).

Single nucleotide variations (SNVs) were filtered using the following criteria: minimum coverage 10, more than 90% of a single different nucleotide to each position. Unreliable SNVs (located in paralogous or highly polymorphic genes, transposons, VNTR regions and deleted regions of a portion of isolates) were excluded to avoid false positive or negative SNVs (S3 Table). Ambiguous SNVs (more than 85% of a single different nucleotide to each position) were also curated (S4 Table).

### Construction of a phylogenetic tree

The topology of a phylogenetic tree based on the 58 SNVs specific to M-strains was verified using Neighbor-joining method and maximum likelihood method under the Jukes–Cantor model using MEGA6 [37]. T85 were used for outgroup rooting.

### Surveillance of M-strain clonal isolates

To find clonal isolates of M-strains, 2,467 clinical isolates of six M. tuberculosis surveillances were screened: Yamagata Pref. (obtained 2009–2011, 186 isolates); Tokyo metropolitan area (obtained 2004–2007, 282 isolates); Osaka City 1 (obtained January 2006 – December 2008, 1,040 isolates); Osaka City 2 (obtained from high-incidence areas January 2006 – December 2010, 295 isolates); Kobe City (obtained January 2009 – December 2010, 449 isolates); Okinawa Pref. (obtained January 2005 – March 2007, 215 isolates). They included five clones of M-strains subjected to genomic comparisons. Spoligotypes [38] or a definitive SNP [39] were analyzed to classify them into Beijing or non-Beijing lineage. Then, types of sequence (ST) of 1,912 isolates belonging to Beijing lineage were verified for assignment to sublineages as described in an earlier report [32]. They are presented in Table 1.

**Table 1:** Clinical isolates of surveillance studies of *Mycobacterium tuberculosis* subjected to screening analysis of M-strains.

**Study**	**Area**	**Period**	**No. of isolates**		
			Total	Beijing	Modern Beijing
Osaka-1	Osaka City, normal areas	2006.1–2008.12	1040	839	223
Osaka-2	Osaka City, high incidence areas	2006.1–2010.12	295	222	89
Okinawa	Okinawa Pref.	2005.1–2007.3	215	147	28
Kobe	Kobe City	2009.1–2010.12	449	353	86
Tokyo	Tokyo, a metropolitan area	2004.6–2007.12	282	217	54
Yamagata	Yamagata Pref.	2009.1–2011.12	186	134	24

### Phylogenetic assignment of clonal isolates of M-strain

The SNV (1757565, G to A) of 504 isolates belonging to the modern Beijing sublineage were determined using real-time PCR system using pairs of MGB probes by LightCycler 480 and FastStart TaqMan Probe Master (Roche Inc.) to find authentic M-strain clones. Newly identified M-strain clones (57 isolates) were further analyzed by real-time PCR or Sanger sequencing for 55 SNVs (except for two drug-resistance related mutations in 6503R). Primers and probes designed for these purposes are presented in S5 and S6 Tables.

### Ethics statement

The Ethics Committee of Institute of Tropical Medicine, Nagasaki University has approved the research protocol (Approval No. 130606112). Consent was not obtained from respective patients directly as the *M*. *tuberculosis* clinical isolates were routinely collected to analyze their genotypes for TB control activity in each local government, following low concerning the prevention of infectious diseases. All samples for this study were anonymized and private information of patients (such as age, gender, living areas, etc.) had been removed prior to the procedures.

## Results

### Comparative genomics of M-strain

**Fig 2:**
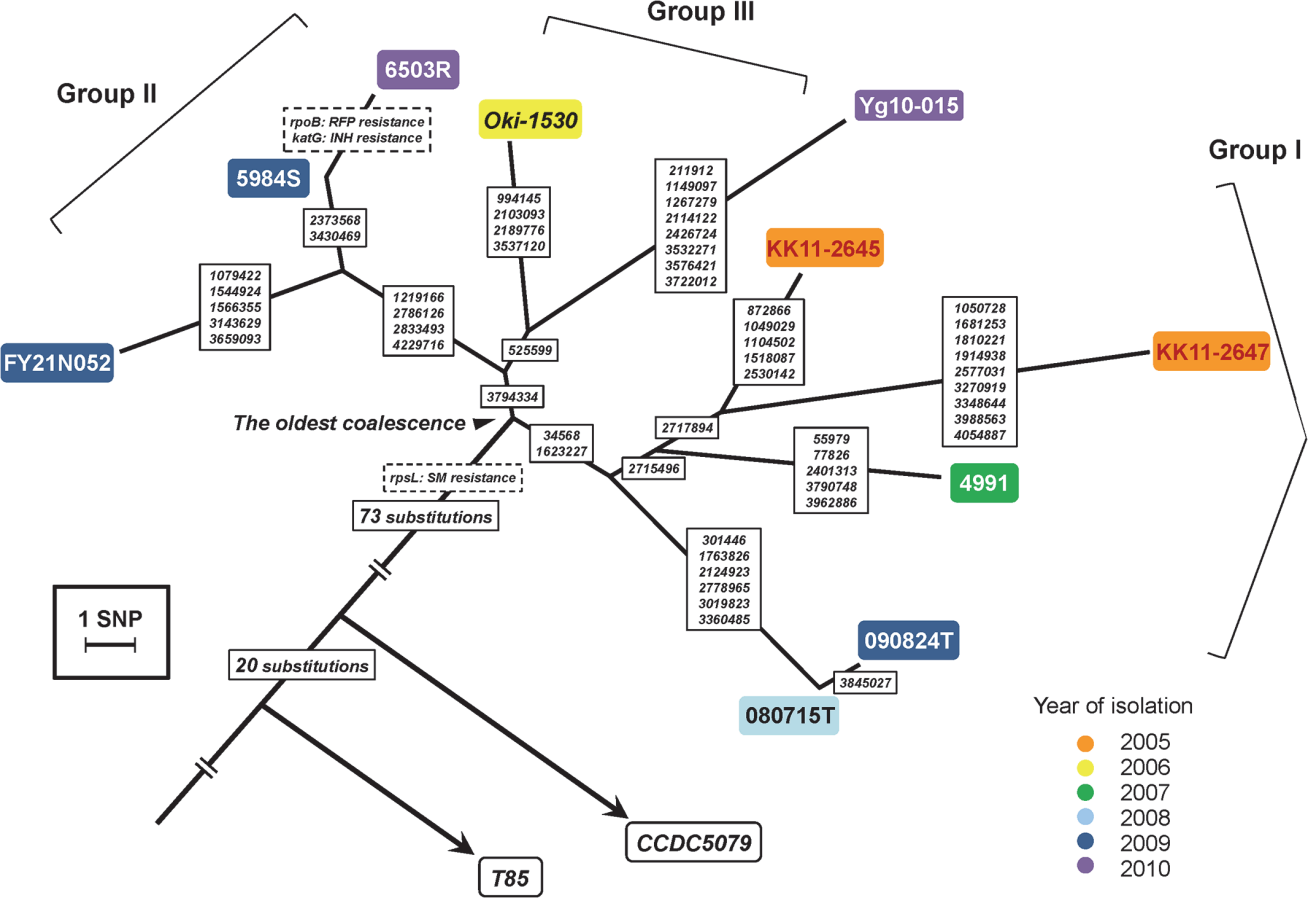
Phylogenetic tree of 10 clonal isolates of M-strain based on SNVs identified from genomic comparison. Two referential strains, T85 and CCDC5079, are also depicted according to the SNVs to H37Rv. The isolates were assigned topologically to three sub-clonal groups (Group I, KK11-2645, KK11-2647, 4991, 080715T, and 090824T; Group II, Oki-1530 and Yg10-015; and Group III, FY21N052, 5984S and 6503R). Positions of SNVs on whole genome sequence of H37Rv (AL123456.2) were put on each branch.

To confirm the clonality of the genotype of M-strain isolated from geographically distant areas, SNVs of 10 isolates and a referential strain T85 were determined by mapping analysis of short reads to the H37Rv genome sequence. Consequently, 1,254–1,261 SNVs per respective isolates on 915 genes and 156 intergenic regions were determined. Of the SNVs of isolates, 151 were screened by differences from T85. They could be separated into two phylogenetic classes: SNVs common to the isolates (94 SNVs, including SM resistance related mutation (*rps*L K43R)) and 57 variable SNVs within the isolates (Fig. 2; mutated positions and genes are listed on S5 and S7 Tables). The read data were also mapped to a complete genome sequence of CCDC5079. Finally, the number of unique SNVs common to the clones of M-strain counted 75. Variable 57 SNVs showed no change.

**Fig 3:**
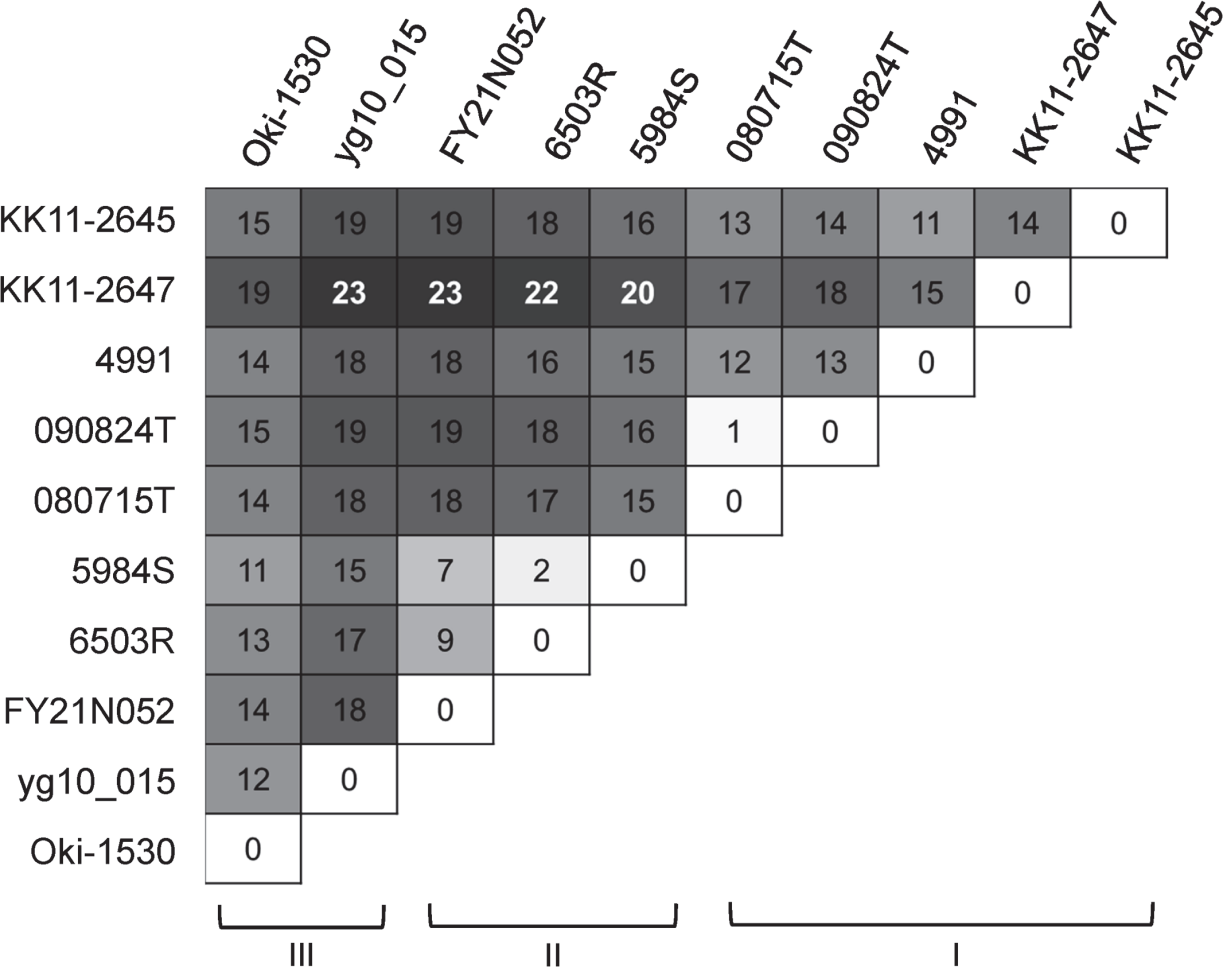
Numbers of SNVs between pairwise isolates of 10 M-strains identified from genomic comparison.

The number of accumulated SNVs from the oldest coalescence (Fig. 2, arrowhead) was 13 at most (KK11-2647). Only one SNV existed between household transmission (090824T and 080715T). Intra-patient isolates exhibited no more than two SNVs in drug resistance genes, *rpo*B and *kat*G (Fig. 2). These numbers of SNVs were correspondent with the previous report describing that the genomic difference might be estimated under 5 SNVs among highly related cases [17]. However, other pairwise comparisons showed 7 to 23 SNVs (Fig. 3).

### Surrogate marker of M-strain

One of 74 bottleneck SNVs common to 10 isolates of M-strain (1757565, G to A) was used as a surrogate marker to detect the authentic clones of M-strain from surveillance studies in five cities. Of 2,467 isolates, 58 were filtered as authentic clones possessing the mutation (Table 2). They included all 38 VNTR-identical types and 13 single locus variant types of M-strains, without exception. It was observed that VNTR genotypes of the isolates fluctuated within 3 of 24 loci at most (6 of double locus variants and 1 of triple locus variant). Deviation was found in various loci, not only in hyper-variable loci, QUB-3232, VNTR3820, and VNTR4120, but also in core loci which were applied to standard subsets [6,12]. They were also all verified as streptomycin resistant (data not shown).

**Table 2:** Clonal isolates of M-strain detected by a new definition of SNV and divergence in their VNTR genotypes.

Sub-clonal group *	**Isolate name**	**Year of isolation**	**Origin**	**ΔVNTR**		**Notes^‡^**
				**Allele No.**	**Locus**	
ND ^†^	4867	2006	Osaka-1	0		
ND	5283	2006	Osaka-1	1	VNTR3336	
ND	5562	2008	Osaka-1	1	Mtub39	
ND	5183	2006	Osaka-2	0		
I	S288	2005	Tokyo	2	QUB-3232, QUB-4120	
I	S349	2005	Tokyo	0		
I	S416	2006	Tokyo	0		
I	S464	2007	Tokyo	2	QUB-11a, QUB-4120	
I	4853	2006	Osaka-1	0		
I	5006	2006	Osaka-1	1	QUB-4156	
I	4991	2007	Osaka-1	0		Subjected to genomic comparison
I	4992	2007	Osaka-1	0		Household case of 4991
I	5120	2007	Osaka-1	0		
I	5614	2008	Osaka-1	0		
I	5997	2008	Osaka-1	0		
I	4671	2006	Osaka-2	0		
I	6421	2008	Osaka-2	1	QUB-26	
I	6604	2009	Osaka-2	0		
I	7086	2010	Osaka-2	0		
I	FY21SPC005	2009	Kobe	1	QUB-4120	
I	Oki-1632	2007	Okinawa	1	QUB-4120	
I	Oki-1637	2007	Okinawa	1	QUB-4120	
I	Oki-1638	2007	Okinawa	1	QUB-4120	
II	4459	2006	Osaka-1	0		
II	4857	2006	Osaka-1	0		
II	4864	2006	Osaka-1	0		
II	4883	2006	Osaka-1	1	VNTR3336	
II	5189	2006	Osaka-1	0		
II	5193	2006	Osaka-1	0		
II	5286	2006	Osaka-1	0		
II	5641	2006	Osaka-1	2	Mtub21, Mtub39	
II	5642	2006	Osaka-1	2	QUB-11b, Mtub24	
II	5217	2007	Osaka-1	0		
II	5391	2007	Osaka-1	1	QUB-3232	
II	6008	2007	Osaka-1	0		
II	6065	2007	Osaka-1	0		
II	6129	2007	Osaka-1	0		
II	6247	2008	Osaka-1	1	QUB-11b	
II	6446	2008	Osaka-1	2	Mtub04, MIRU26	
II	5491	2007	Osaka-2	0		
II	5631	2008	Osaka-2	0		
II	6416	2008	Osaka-2	1	Mtub21	
II	5984S	2009	Osaka-2	0		Subjected to genomic comparison
II	7259	2010	Osaka-2	3	Mtub24, MIRU26, ETRA	
II	7264	2010	Osaka-2	1	Mtub39	
II	FY21N052	2009	Kobe	0		Subjected to genomic comparison
II	FY21SPC002	2009	Kobe	0		
II	FY22KIH052	2010	Kobe	0		
III	yg2010-015	2010	Yamagata	0		Subjected to genomic comparison
III	S372	2006	Tokyo	1	QUB-3232	
III	S449	2007	Tokyo	0		
III	S463	2007	Tokyo	0		
III	4666	2006	Osaka-1	0		
III	4826	2006	Osaka-1	0		
III	5053	2006	Osaka-1	0		
III	5116	2007	Osaka-1	0		
III	5936	2007	Osaka-1	0		
III	Oki-1530	2006	Okinawa	0		Subjected to genomic comparison

**Fig 4:**
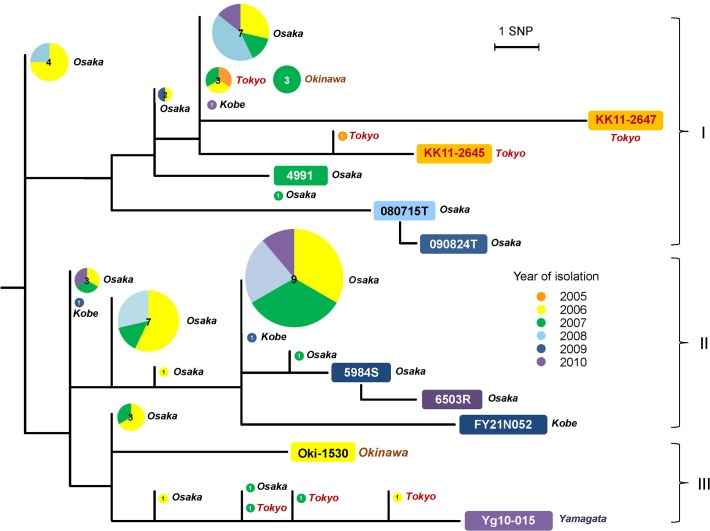
Phylogenetic positions of 53 clonal isolates of M-strains identified using a surrogate SNV marker (position 1757565, G to A). The tree is topologically identical to Fig. 2. Circles on the tree denote clones isolated from respective areas (Yamagata, Tokyo, Osaka, Kobe, and Okinawa). Sizes and numbers of circles show the number of clones. Colours in the circles represent years of isolation.

* Determined by a phylogenetic tree illustrated in Fig. 4.

† Not determined. Four isolates were not assigned to sub-clonal groups because they possessed no SNVs identified by genomic comparison.

‡ Some isolates subjected to genomic comparison were absent from the table, because they were cultured from different studies. Two household isolates in Osaka (080715T and 090824T) were cultured for transmission tracking. Two independent isolates in Tokyo (KK11-2645 and KK11-2647) were cultured and stocked for other purposes in a hospital. An MDR isolate 6301R had been excluded from the surveillance study Osaka-2, because the identical patient of 5984S was found in the surveillance study Osaka-2 in 2009.

### Phylogenetic distribution of M-strain clonal isolates

To evaluate the SNV list derived from comparative genomics as an estimation tool of nationwide transmission history of M-strains, 55 of 57 SNVs (excluding two drug-resistance related mutations of 6503R) were verified for each clone systematically by direct sequencing. Variable accumulation of SNVs was observed in the isolates (Fig. 4). The isolates were distributed in three phylogenetic sub-clonal groups with a bias of origins (Fig. 4 and Table 2). In group II, origins of isolates were exclusively two nearby areas: Osaka and Kobe. In contrast, groups I and III included isolates having various origins such as Yamagata, Tokyo, and Okinawa. Four isolates were unable to be classified into the subgroups because of a lack of SNVs. To avoid the possibility that these four isolates were overestimated as clones by the SNV 1757565, remaining 10 intergenic SNVs on the bottleneck mutations of the strain (S7 Table) were verified by direct sequencing using primers reported previously [40]. Consequently, these intergenic positions of the isolates were determined as variant nucleotides (data not shown).

## Discussion

Genotyping with high discriminatory power is necessary to detect unnoticed transmission of *M*. *tuberculosis* among patients. In actuality, VNTR genotyping provides high resolution among strains, but unexpected concordance/discordance can occur in some cases because VNTR alleles are reversible; then backward mutations occasionally occur. To ascertain the precise transmission contexts of TB, accumulating nucleotide mutations throughout the whole genome sequences of clinical isolates may take the place of existing genotyping tools. In this study, we specifically examined a nationwide genotype strain in Japan: M-strains. The inexplicable emergence of the strain might affect the valid interpretation of genotypic identification to find transmission routes. It was therefore important to elucidate a scenario of nationwide detection of the strain to maintain a legitimate concept of genotypic surveillance of *M*. *tuberculosis*.

In the genomic comparison, detected SNVs between household cases and recurrence of a single patient was few (one SNV in a household case, 080715T and 090824T, and two drug resistance related SNVs in a single patient, 5984S and 6503R). This result was consistent with a previous large-scale study [17]. In contrast to them, other pairwise comparisons showed at least seven SNVs (5984S and FY21N052) (Fig. 3). These results correspond with no epidemiological links among the eight clonal isolates of M-strains, even in geographically related cases (Tokyo and Osaka/Kobe). Such marked differences of the numbers of SNVs between direct and indirect transmission might support the possibility of genomic comparison to discriminate precise transmission with long distance, which might be difficult using existing protocols [41, 42].

In our study, genomic variation retrieved from 10 isolates of M-strain could provide unprecedented information related to strains, such as their bottleneck (78 SNVs, Fig. 2) and genotypic markers for refined discrimination (55 instra-strain SNVs, Fig. 4). Clonal isolates of M-strain determined by a surrogate SNV marker could be assigned to sub-clonal groups by the 55 intra-strain SNVs. This result signifies that the single SNV could sort out the authentic clone from isolates collections accurately. SNV-based screening of certain strains can enhance the accurate detection of clonal isolates in surveillance studies of *M*. *tuberculosis* in comparison to VNTR genotyping only. The genotypic robustness can overcome fluctuating VNTR typing, once determined based on genomic comparison. Such a strategy depends on a nature of *M*. *tuberculosis* complex that genomic recombination is rare in its evolution, which supports low frequency of homoplastic SNVs in the intra-species phylogeny [43]. It may be helpful to assess the actual spread of dangerous strains such as those of transmissible MDR strains, from surveillance studies conducted for the molecular epidemiology of TB.

The phylogenetic assignment of clonal isolates by SNVs offered a clue to separate them into local epidemic cases (sub-clonal group II) and nationwide expansion (sub-clonal group I and III) (Fig. 4). Such a subdivision of clonal isolates may help to elucidate ongoing outbreaks in local settings reasonably, although our study could not pick out SNV accumulation of each authentic clone precisely, owing to the restricted number of isolates for genomic comparison. Moreover, isolates groups with identical SNVs can be estimated as close transmission cases more probably than other isolates. These SNVs are therefore useful as genetic markers to refine the clone transmission history for future surveillance. Such feedback from genomic comparison to genetic markers may be an efficient means of utilizing of deep sequencing data of clinical isolates in various settings. Genomic comparison of recent studies has also emphasized discrimination of identical genotypic isolates within various situations [16,18,20–22]. The thorough technique might come to play a more important role in practical epidemiological fields of *M*. *tuberculosis* when it becomes applicable to surveillance studies representing geographical/provincial settings of TB.

## Supporting Information

S1 Table A definition of M-strains based on 24 loci VNTR genotyping method. (XLSX)

S2 Table Summary of short reads of 10 clonal isolates of M-strains. (XLSX)

S3 Table ORFs and intergenic regions from which called substitution mutations were excluded from genomic comparison in this study. (XLSX)

S4 Table Curated SNVs determined by mapping analysis of short reads to the H37Rv genomic sequence. (XLSX)

S5 Table Positions of SNVs within clonal variation of M-strains identified by mapping analyses and primer sequences for direct sequencing. (XLSX)

S6 Table Sequences of primers and probes for real-time PCR to verify SNVs of M-strains. (XLSX)

S7 Table SNVs common to 10 clonal isolates of M-strain identified by their difference from a referential strain T85. (XLSX)
